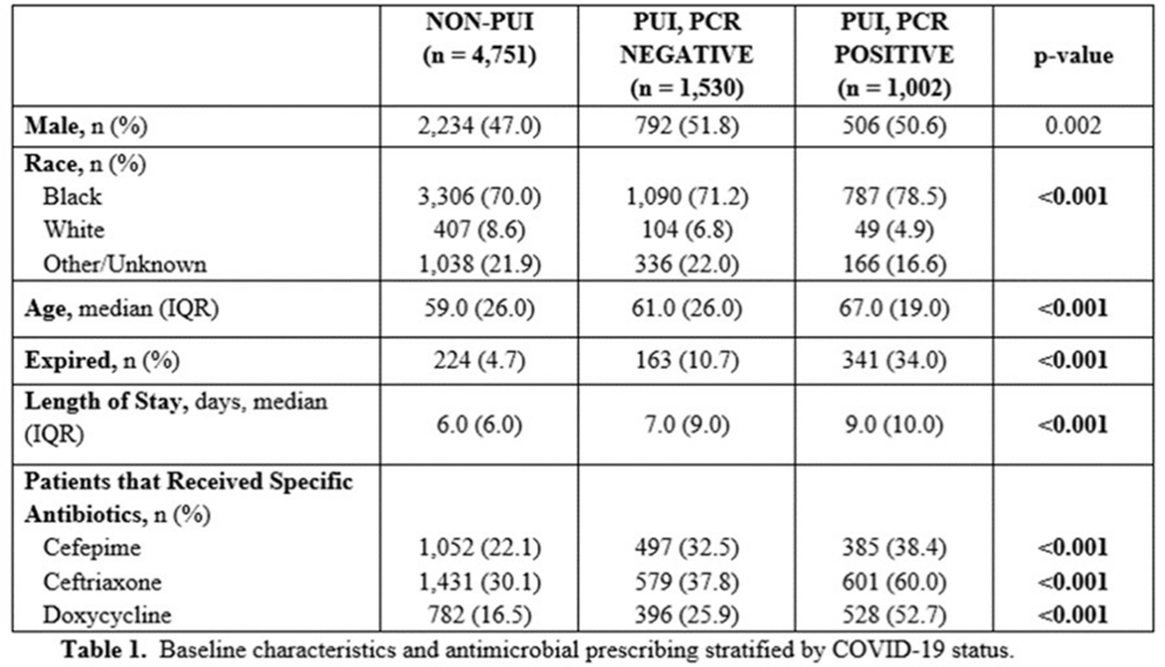# How the COVID-19 Pandemic Affected Antimicrobial Prescribing Practices at a Tertiary-Care Healthcare System in Detroit, Michigan

**DOI:** 10.1017/ash.2021.69

**Published:** 2021-07-29

**Authors:** Angela Beatriz Cruz, Jennifer LeRose, Avnish Sandhu, Teena Chopra

## Abstract

**Background:** Inappropriate antimicrobial use continues to threaten modern medicine. The ongoing pandemic likely exacerbated this problem because COVID-19 presents similarly to bacterial pneumonia, confusion exists regarding treatment guidelines, and testing turnaround times (TATs) are slow. Our primary object was to quantify antimicrobial use changes during the pandemic to rates before the crisis. A subanalysis within the COVID-19 cohort was completed based on SARS-CoV-2 status. **Methods:** The pre–COVID-19 period was January–May 2019 and the COVID-19 period was January–May 2020. Subanalyses were used to explore differences in antibiotics use between persons not under investigation (non-PUIs), SARS-CoV-2–negative PUIs, and SARS-CoV-2–positive PUIs. Non-PUI patients were those without respiratory symptoms and/or fever. The χ^2^ and Wilcoxon signed rank-sum tests were used for analysis. **Results:** During the 2019 and 2020 study periods, 7,909 and 7,283 patients received >1 antimicrobial, respectively (Figure 1). Overall, antibiotic therapy per 1,000 patient days increased from 633.1 before COVID-19 to 678.5 during COVID-19, a 7.2% increase (Table 1). Notably, broad-spectrum respiratory antibiotics demonstrated a significant increase between pre–COVID-19 and COVID-19 cohorts (p < 0.001). Of the 7,283 patients within the COVID-19 cohort, 34.7% (n = 2,532) were PUI and 13.8% (n = 1,002) of these patients tested SARS-CoV-2 positive. Again, broad-spectrum respiratory antibiotics use was significantly increased for COVID-19 patients (p < 0.001). Of note, the proportion of patients receiving respiratory antibiotics steadily decreased over time (R^2^ = 0.99). **Conclusions:** There was a significant increase in antibiotic use during the COVID-19 pandemic. Encouragingly, antimicrobial use decreased over time, likely due to (1) faster TATs, (2) real-time education to clinicians and subsequent de-escalation of unnecessary antimicrobials, and (3) development of treatment guidelines as new research emerged.

**Funding:** No

**Disclosures:** None

**Figure 1.**
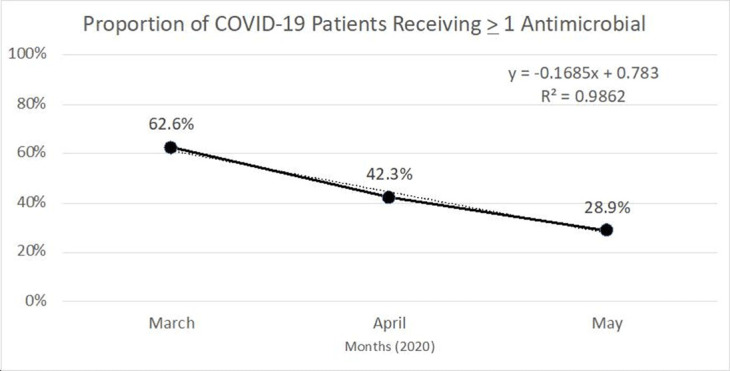

**Table 1.**